# Enhanced expression of long non-coding RNA NEAT1 is associated with the progression of gastric adenocarcinomas

**DOI:** 10.1186/s12957-016-0799-3

**Published:** 2016-02-24

**Authors:** Yanling Ma, Li Liu, Fei Yan, Wujie Wei, Jie Deng, Jianhai Sun

**Affiliations:** Department of Oncology of Zhongshan Hospital, Wuhan University, No. 26 Zhongshan Road, Wuhan, Hubei 430033 China

**Keywords:** Gastric adenocarcinoma, Long non-coding RNA, NEAT1, Cell proliferation

## Abstract

**Background:**

Long non-coding RNAs (lncRNAs) are emerging as new players in the cancer. The aim of this study was to examine the abnormalities of NEAT1 (nuclear paraspeckle assembly transcript 1, also known as MENε/β) in gastric adenocarcinomas (GACs).

**Methods:**

One hundred thirty-one GAC tissues and matched adjacent normal tissues (ANTs) were collected from patients who undergone surgery. Differences in of NEAT1 expression were examined via quantitative reverse transcriptase PCR (qRT-PCR). WST-1 assay and transwell assay were carried out in vitro to investigate the proliferation and migration of GAC cells with alteration in NEAT1 long non-coding RNA (lncRNA) expression.

**Results:**

The expression levels of lncRNA NEAT1 were significantly elevated in GAC tissues (*P* < 0.001) compared with ANTs. There was also a statistical difference in NEAT1 expression between early and advanced GACs (*P* = 0.0111). GACs with lymph node metastasis (LNM) expressed higher levels of NEAT1 lncRNA compared with those without LNM (*P* = 0.004). In the in vitro experiments, the proliferation but not migration of GAC cells was attenuated after NEAT1 knockdown by RNA interference.

**Conclusions:**

Expression of NEAT1 lncRNA was enhanced in GACs; and NEAT1 may influence GAC progression by promoting tumor growth.

## Background

Gastric cancer (GC) is a major health burden throughout the world. It is currently the most common cancer in China, responsible for about 300,000 deaths per year [1, 2]. GC is commonly diagnosed at an advanced stage because of the lack of early detection strategies and is usually associated with a dismal outcome [3, 4]. Most of GCs are adenocarcinomas. The development of gastric cancer is a complex, multistep process involving multiple genetic and epigenetic alterations of oncogenes, tumor suppressor genes, DNA repair genes, cell cycle regulators, and signaling molecules [5–7].

Recent studies have highlighted the role of a group of long (>200 bp) non-coding RNAs (lncRNAs) in carcinogenesis and suggested that this class of genes might be used as biomarkers in cancer [8–10]. LncRNAs are pervasively transcribed in the genome and are emerging as new players in tumorigenesis due to their various functions in transcriptional, posttranscriptional, and epigenetic mechanisms of gene regulation [11]. LncRNAs are dysregulated in a number of cancers, in which they demonstrated both oncogenic and tumor-suppressive roles. These evidences suggested that lncRNAs’ aberrant expression might be a substantial contributor in cancer development. Several cancer-related long non-coding RNAs have been reported to be correlated with GAC proliferation or metastasis, including HOTAIR, ANRIL, MRUL, CCAT1, NEAT1 and HULC, etc. [12, 13].

However, there is few report about the expression of nuclear-enriched abundant transcript 1 (NEAT1) lncRNA in GCs. NEAT1 is a novel long non-coding RNA (lncRNA) which serves as a crucial regulator in several cancers [14, 15]. NEAT1 had higher expression in the hepatocellular carcinoma (HCC), which promoted the clinical features of HCC [16]. NEAT1 might also be associated with tumorigenesis and progression in non-small cell lung cancers (NSCLC) [17]. NEAT1 drove oncogenic growth in prostate cancer [18]. We asked whether there were abnormalities of this lncRNA in GCs.

In the present study, we examined the expression levels of NEAT1 lncRNA in collected GAC and ANT samples and found that the expression levels of NEAT1 lncRNA were significantly enhanced in GACs and had an association with tumor stages. Knockdown of NEAT1 lncRNA led to impaired proliferation of GAC cells in vitro.

## Methods

### Patients and tissue collection

GAC samples were obtained from 131 surgical patients of the Department of Gastroenterology, Zhonghan Hospital of Hubei Province. All 131 tumors were adenocarcinomas and other types of tumors (squamous cell, carcinoid, and stromal tumors) were excluded. A summary of cohort characteristics was shown in Table 1. Case-matched adjacent normal mucosa, located at least 3 cm far from the macroscopically unaffected margins of the tumor, were defined as normal controls. All the samples were stored in liquid nitrogen immediately after the operations. Gastric carcinomas were staged according to the TN classification system and graded into five groups: stage 0 (TisN0M0, *n* = 0), stage 1 (T1N0–1M0, *n* = 4), stage 2 (T1N2M0, T2N0–1M0, *n* = 30), stage 3 (T2N2M0, T3N1–2M0, T4N0M0, *n* = 66), stage 4 (T4N1–2M0,T1–3N3M0, TanyNanyM1, *n* = 31). Matched samples of GACs and adjacent normal tissues (ANTs) were immediately stored in liquid nitrogen after operation. For each sample, a portion of tissue was disposed to make frozen sections and then micro-dissected by the Eppendorf Microdissection System (Siskiyou MX160L micromanipulator and PixeLink PL-A662 color CCD firewire camera). Then, the dissected tissues were subjected to real-time PCR analysis. All patients were informed about the aims of specimen collection and gave signed written consent in accordance with the ethical guidelines of Zhongshan University. The investigations were conducted according to the Declaration of Helsinki principles and the study was approved by the ethical committee of Zhongshan Hospital of Hubei Province (EC201402348).

**Table 1:** Summary of the cohort characteristics

Characteristics	Information	
Gender	Female	59
	Male	72
Age	Average age	51.2
	Range	23~75
HPV	High risk (HPV16, HPV18, or both)	99
	Other HPVs	37
Lymph node metastasis (LNM)	With LNM	33
	Without LNM	103
Smoking history	Yes	55
	No	76

### RNA extraction and real-time PCR

Total RNA was isolated from tissues by using AxyPrepTM Blood Total RNA MiniPrep Kit (Axygen) according to the manufacturer’s instruction. First-strand complementary DNA (cDNA) was synthesized with RevertAid™ First Stand cDNA Synthesis Kit (Fermentas) using a random hexamar primer. Quantitative PCR was performed through BioRad Chromo4 real-time PCR system. The primer sets for amplifying NEAT1 and reference gen (ACTB) are the following: NEAT1-F: TGG ACT AGCT CAG GGA CTT CAG; NEAT1-R: TCT CCT TGA CCA AGA CTT CCT TC; ACTB-F: GAC CTG ACT GAC TAC CTC ATG AAG AT; ACTB-R: GTC ACA CTT CAT GAT GGA GTT GAA GG. At the end point of PCR cycles, melt curves were made to check product purity. The level of NEAT1 was expressed as a ratio relative to the β-actin messenger RNA (mRNA) in each sample. Exploratory data analysis using scatter plot was applied to visually identify the expression level of target mRNA. Statistical analysis was performed using paired *t* test or non-parametric test. *P* values less than 0.05 were considered statistically significant.

### Cell culture

Human gastric cancer cell lines MKN-45, BGC823, MGC803, SGC7901, AGS, and MKN28 were obtained from the Cobioer Biosciences Co., Ltd. (Shanghai, China) where they were characterized by mycoplasma detection, DNA fingerprinting, isozyme detection, and cell vitality detection. These cell lines were purchased in August 2014 and immediately expanded and frozen such that they could be restarted every 3 to 4 months from a frozen vial of the same batch of cells. MKN28 cells were cultured in Dulbecco’s modified Eagle’s medium (DMEM, Gibco) supplemented with 10 % fetal bovine serum (PAA) and 1 % penicillin/streptomycin (Life Technologies, Inc.).

### Reagents and cell transfection

Lipofectamine 2000 transfection reagent (Invitrogen, Carlsbad, CA, USA) was used. The pcDNA3.1-NEAT1 and empty vector (used as a negative control) was purchased from Invitrogen, Shanghai, China. Cells were seeded in 96 plates 24 h before the experiment. MGC803 cells were transfected with pcDNA3.1-NEAT1 or negative control. The MGC803 was transfected.

### NEAT1 knockdown by lentiviruses

NEAT1-set small interfering RNA (siRNA)/small hairpin RNA (shRNA)/RNAi Lentivector as well as control shRNA vector were purchased from Nanjing EnoGene Biotechnology Limited Corporation (catalog no. ES000103). To generate lentiviruses expressing MALAT-1 shRNA and control shRNAs, HEK293T cells grown on a 10-cm dish were transfected with 6 μg of NEAT1-set shRNA lentivector or control vector, 6 μg of pREV, 6 μg of pGag/Pol, and 2 μg of pVSVg. Twelve hours after transfection, cells were cultured with DMEM medium containing 20 % FBS for an additional 36 h. The culture medium containing lentivirus particles was centrifuged at 10,000 ×g for 2 min and then used for infection. Twenty-four hours after infection, cells were cultured with fresh medium for another 24 h, followed with further experiment. The knockdown efficiency was evaluated by real-time PCR analysis.

### Cell proliferation assay

Cell proliferation was measured by WST-1 assay. Cells were plated in 96-well culture plates (1 × 10^3^ per well); WST-1 (Roche) assay measuring the activity of mitochondrial dehydrogenases was performed following the manufacturer’s instruction at 0-, 1-, 2-, 3-, 4-, and 5-day time points.

### Cell migration assay

Migration assays were performed using 24-well transwell units with 8-mm pore size polycarbonate inserts (BD Biosciences). Transwells were coated overnight with 10 mg/mL of fibronectin in phosphate-buffered saline at 48.8 °C, followed by incubation with 1 % bovine serum albumin for 1 h at 37 °C. The MKN45 and AGS cells transfected with NEAT1 shRNA or nonsense strand were detached with trypsin/ethylene diamine tetraacetic acid, washed once with DMEM containing 10 % fetal bovine serum (FBS), and re-suspended in DMEM containing 1 % FBS at 2 × 10^5^ cells/mL. Aliquots (100 mL) of cell suspensions were directly added to the upper side of each chamber. Following incubation for 12 h, the cells on the upper side of the membrane were removed, whereas the cells that migrated to the underside were fixed with 3 % formaldehyde and stained with 0.3 % crystal violet for 10 min. The number of cells on the underside of the membrane was counted in five different fields with a light microscope at 20 °C, and the mean and standard deviation were calculated from three independent experiments.

### Statistical analysis

GraphPad Prism software (Version 5.0) was used to analyze the obtained data. Results of the NEAT1 lncRNA expression for paired GACs and ANTs or paired GACs and local lymph node metastases were compared using paired *t* test. Results of the NEAT1 lncRNA expression in different GAC groups were compared using non-parametric Mann–Whitney test. *P* values less than 0.05 were considered statistically significant.

## Results

### Increased expression of NEAT1 lncRNA in GACs

**Fig. 1:**
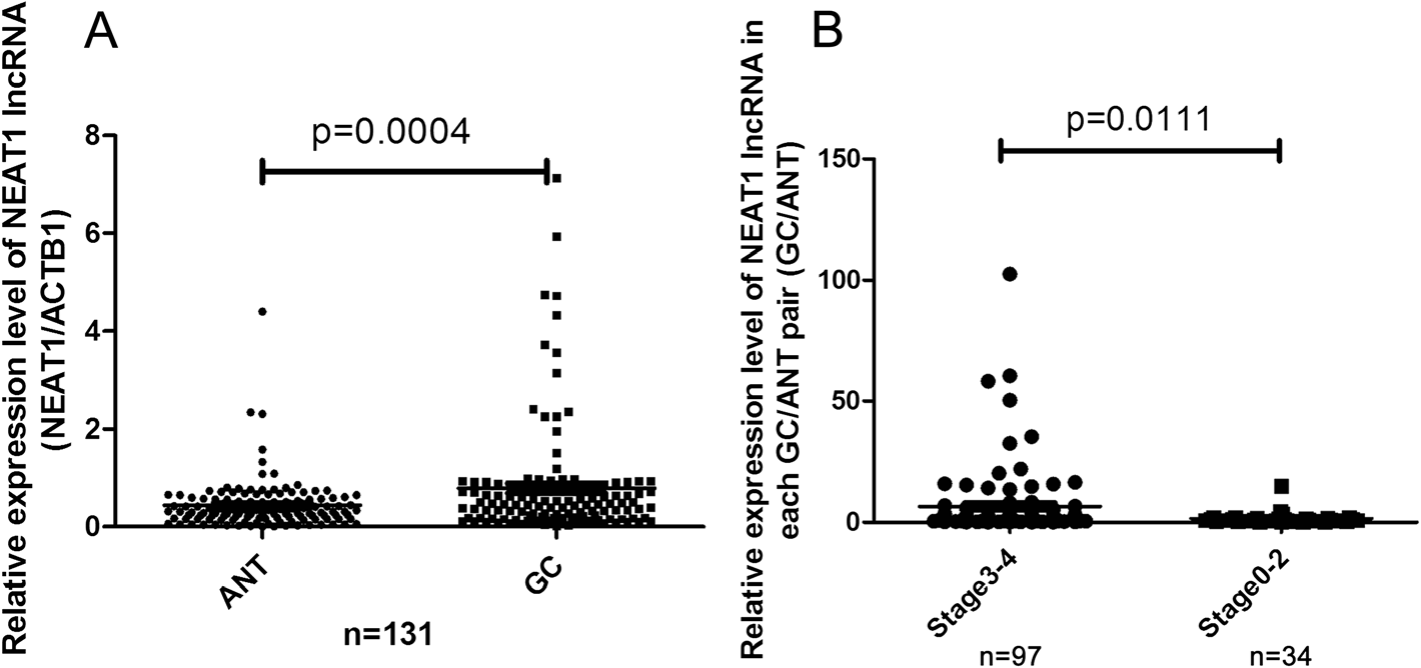
Enhanced expression of NEAT1 lncRNA in GAC. Real-time PCR assay was carried out as described under the “Methods” section, and the results were obtained from indicated group of samples. **a** Scatter plot illustrated the relative expression of indicated lncRNAs as a ratio of lncRNA to β-actin mRNA in each sample. **b** Scatter plot illustrated the relative expression of NEAT1 as a ratio of GAC to ANT mRNA (GAC/ACTB: ANT/ACTB) in each sample

The expression levels of NEAT1 lncRNA in the collected GAC samples were examined using real-time PCR assay. As shown in Fig. 1a, the expression levels of NEAT1 lncRNA were markedly enhanced in GACs compared to ANTs (*P* < 0.001). There was a statistical difference between the group of early-stage GACs and advanced GACs (*P* < 0.01). This result was in line with the reported findings in NSCLCs and HCCs [16, 17].

### An association between NEAT1 expression and lymph node metastasis

**Fig. 2:**
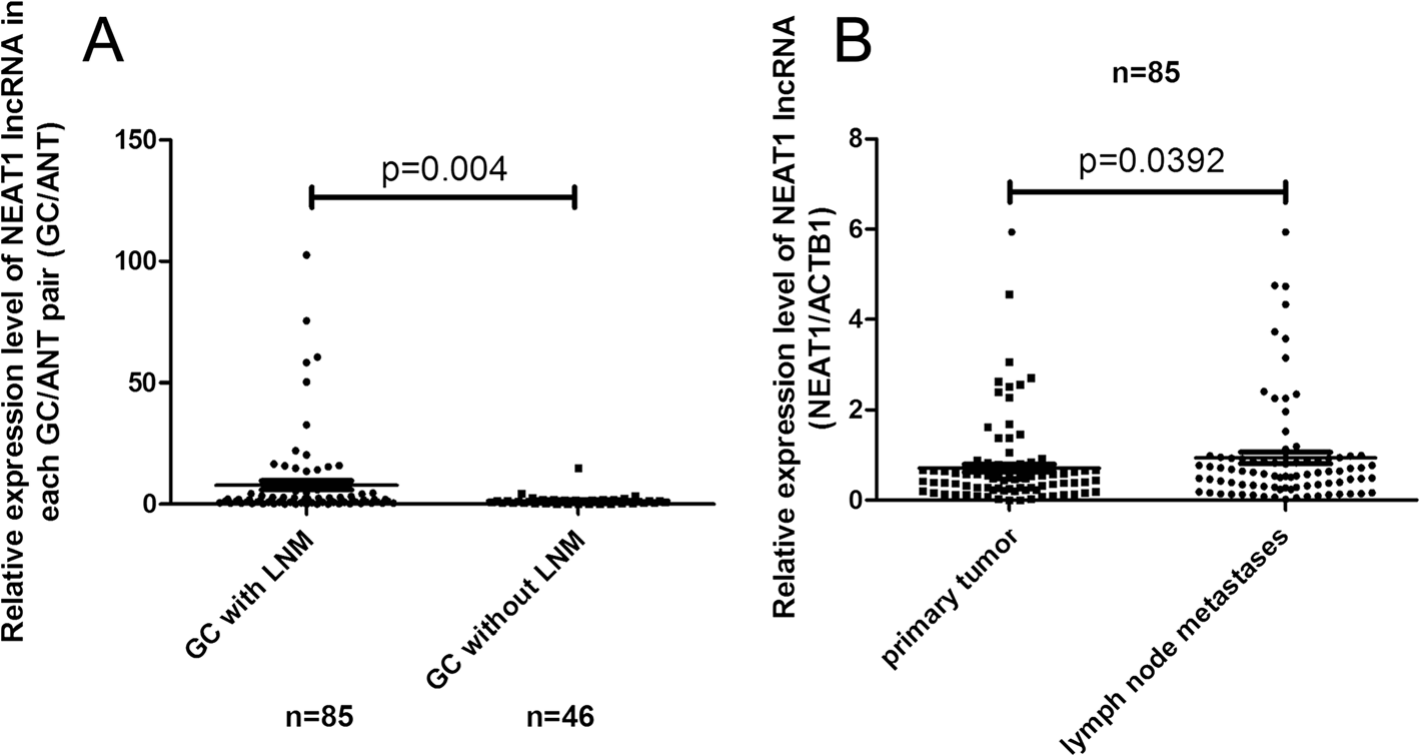
Higher expression of NEAT1 in GAC with lymph node metastasis. **a** Scatter plot illustrated the relative expression of NEAT1 as a ratio of GAC to paired ANT in the GACs with or without lymph node metastasis. **b** Scatter plot illustrated the relative expression of NEAT1 as a ratio of GAC to paired ANT in the primary tumors and lymph node metastases

Next, we compared the NEAT1 lncRNA expression between tumor groups with (*n* = 85) or without lymph node metastasis (*n* = 46). As shown in Fig. 2a, there was a marked difference of NEAT1 expression between GAC with or without lymph node metastasis (LNM) (*P* = 0.004). In the GACs with LNM (*n* = 85), we also compared the expression of NEAT1 lncRNA in primary tumors and metastases. As shown in Fig. 2b, the analysis using paired *t* test showed a minor but significant increase of NEAT1 lncRNA expression in the metastases compared to the corresponding primary tumors (*P* = 0.0392). The association between NEAT1 expression and GAC metastasis might need further investigation.

### Increased expression of NEAT1 lncRNA promotes growth of GAC cells in vitro

**Fig. 3:**
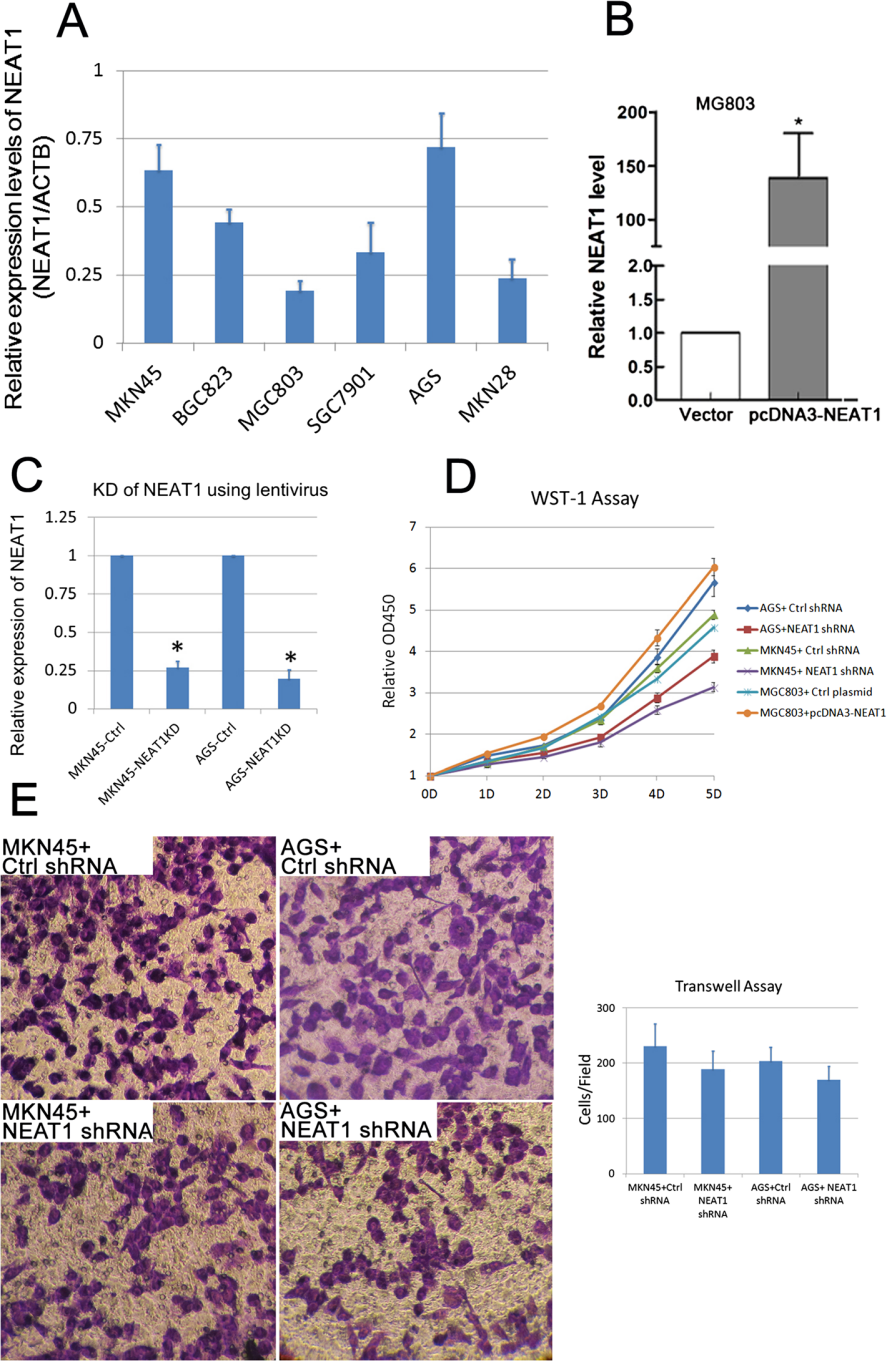
Knockdown of NEAT1 lncRNA impairs growth of GAC cells in vitro. **a** Expression levels of NEAT1 lncRNA were examined by real-time PCR. **b** The expressions of NEAT1 mRNA in MGC803 cells transfected with pcDNA3-NEAT1 and control vector detected by RT-qPCR. **c** After treatment of lentiviruses expressing NEAT1 shRNA and control shRNAs, the expression levels of NEAT1 lncRNA were examined by real-time PCR. The relative expression of NEAT1 lncRNA (as the ratio of NEAT1 lncRNA to β-actin mRNA) is illustrated as a ratio to control (cells transfected with control shRNA). **d** WST-1 (Roche) assay measuring the activity of mitochondrial dehydrogenases was performed following the manufacturer’s instruction at 0-, 1-, 2-, 3-, and 4-day time points. *Error bars* represent the standard deviation of the mean. **e** Cell migration was determined using a transwell assay as described in the “Methods” section. Microscopic image of migrated MKN45 and AGS cells, cells transfected with nonsense strand or NEAT1 shRNA, respectively. Original magnification 200×. The diagrams of migrating cells from the different transfectants are also shown, which come from more than three independent experiments

It was reported that increased NEAT1 lncRNA expression could drive oncogenic growth by altering the epigenetic landscape of target gene promoters to favor transcription [18]. To further investigate the role of NEAT1 lncRNA in GAC progression, in vitro studies using GAC cell lines were performed. We first compared the expression of NEAT1 lncRNA among six cell lines including MKN45, BGC823, MGC803, SGC7901, AGS, and MKN28. As shown in Fig. 3a, MKN-45 and AGS showed relatively higher expression of NEAT1, which were utilized for the following knockdown experiments.

The efficiencies of overexpression and knockdown of NEAT1 in GAC cells were shown in Fig. 3b, c. Then, WST-1 assay was carried out to evaluate the influence of altered expression of NEAT1 on GAC cell proliferation. As shown in Fig. 3d, down-regulation of NEAT1 lncRNA significantly impaired the proliferation of MKN45 and AGS cells, while overexpression of NEAT1 lncRNA resulted in enhanced proliferation in MGC803 cells.

The migration ability of MKN45 and AGS cells transfected with NEAT1 shRNA or scramble RNA was evaluated by transwell assay. We found that the MKN45 as well as AGS cells with decreased expression of NEAT1 showed slightly impaired migration compared with controls (Fig. 3e). Thus, altered expression NEAT1 lncRNA might have minor effect on GAC cells’ migration.

## Discussion

LncRNA contributes significantly to human transcriptome and plays a critical role in cancer development [19]. Here, we focused on the expression of NEAT1 lncRNA in GACs and used a large number of cases in the current study to find a novel correlation between elevated expression of NEAT1 lncRNA and GAC pathogenesis. We also found that advanced GACs had a higher expression of NEAT1 lncRNA than those at early stages, which indicated potential role of NEAT1 abnormalities in GAC progression. A marked difference was observed between GACs with and without LNM, indicating a possible role of NEAT1 lncRNA in the metastasis of GACs. However, in the GAC cases with LNM, the metastases of GACs showed only minor NEAT1 elevation compared to the primary tumors. Thus, the role of NEAT1 lncRNA in the metastasis of GACs needs further investigation.

The key feature of non-coding RNAs (ncRNAs) is that they are not translated into proteins but rather function directly at the RNA level [20, 21]. To investigate the role of NEAT1 abnormalities in GAC, we performed in vitro studies to examine the influences of altered NEAT1 expression on proliferation and migration of GAC cells. We found that knockdown of NEAT1 lncRNA led to a significant impairment of growth in GAC cells, while it had little influence on migration of GAC cells in vitro. These indicate that overexpression of NEAT1 lncRNA in GAC might mainly regulate the genes that correlated with cell growth but not the genes associated with cancer metastasis. These were not fully in accordance with the previous findings in NSCLC [17] or HCC [16], in which the proliferative, migrational, and invasive behaviors of cancer cells were all enhanced by exogenous NEAT1 expression in vitro*.* This discrepancy might be explained by the different expression pattern of mRNAs that were influenced by NEAT1 in various types of cancer. And the mRNAs that were regulated by NEAT1 lncRNA in GAC need to be further investigated using high-throughput assays like microarrays and RNA sequencing.

In conclusion, our findings suggest that the expression level of NEAT1 has the potential to predict GAC progression. However, the functional consequences of altered NEAT1 expression and the underlying mechanisms of the heterogeneous expression levels in GACs need to be further investigated in the future.

## Conclusions

Here, we report a novel finding about the abnormal NEAT1 lncRNA expression in gastric adenocarcinomas (GACs). Our study indicated that increased expression of NEAT1 lncRNA in GACs correlated with tumor stage as well as lymph node metastasis in clinical GAC samples. Knockdown of NEAT1 lncRNA in GAC cells led to impaired growth. Thus, we conclude that enhanced NEAT1 lncRNA may play a role in the progression of GAC pathogenesis.
